# Prevascularized spongy-like hydrogels maintain their angiogenic potential after prolonged hypothermic storage

**DOI:** 10.1016/j.bioactmat.2024.02.035

**Published:** 2024-03-28

**Authors:** Sara Freitas-Ribeiro, Helena Moreira, Lucília P. da Silva, Jennifer Noro, Belém Sampaio-Marques, Paula Ludovico, Mariana Jarnalo, Ricardo Horta, Alexandra P. Marques, Rui L. Reis, Rogério P. Pirraco

**Affiliations:** a 3B's Research Group, I3Bs – Research Institute on Biomaterials, Biodegradables and Biomimetics, University of Minho, Headquarters of the European Institute of Excellence on Tissue Engineering and Regenerative Medicine, AvePark, Parque de Ciência e Tecnologia, Zona Industrial da Gandra, 4805-017, Barco, Guimarães, Portugal; b ICVS/3B's–PT Government Associate Laboratory, Braga/Guimarães, Portugal; c Life and Health Sciences Research Institute (ICVS), School of Medicine, University of Minho, Braga, Portugal; d Department of Plastic and Reconstructive Surgery, and Burn Unity, Centro Hospitalar de São João, Porto, Portugal; e Faculty of Medicine - University of Porto, Portugal

**Keywords:** Tissue engineering, Prevascularization, Hypothermic preservation, Clinical translation

## Abstract

The chronic shortage of organs and tissues for transplantation represents a dramatic burden on healthcare systems worldwide. Tissue engineering offers a potential solution to address these shortages, but several challenges remain, with prevascularization being a critical factor for in vivo survival and integration of tissue engineering products. Concurrently, a different challenge hindering the clinical implementation of such products, regards their efficient preservation from the fabrication site to the bedside. Hypothermia has emerged as a potential solution for this issue due to its milder effects on biologic systems in comparison with other cold preservation methodologies. Its impact on prevascularization, however, has not been well studied. In this work, 3D prevascularized constructs were fabricated using adipose-derived stromal vascular fraction cells and preserved at 4 °C using Hypothermosol or basal culture media (α-MEM). Hypothermosol efficiently preserved the structural and cellular integrity of prevascular networks as compared to constructs before preservation. In contrast, the use of α-MEM led to a clear reduction in prevascular structures, with concurrent induction of high levels of apoptosis and autophagy at the cellular level. In vivo evaluation using a chorioallantoic membrane model demonstrated that, in opposition to α-MEM, Hypothermosol preservation retained the angiogenic potential of constructs before preservation by recruiting a similar number of blood vessels from the host and presenting similar integration with host tissue. These results emphasize the need of studying the impact of preservation techniques on key properties of tissue engineering constructs such as prevascularization, in order to validate and streamline their clinical application.

## Highlights

• Hypothermosol retains structural and cellular integrity of prevascular networks.
• α-MEM induces increased cellular apoptosis and autophagy.
• *In vivo* construct angiogenic potential was retained with hypothermosol.
• Tailored preservation solutions effectively mitigate hypothermia-induced prevascular network damage.

## Graphical abstract

**Figure:**
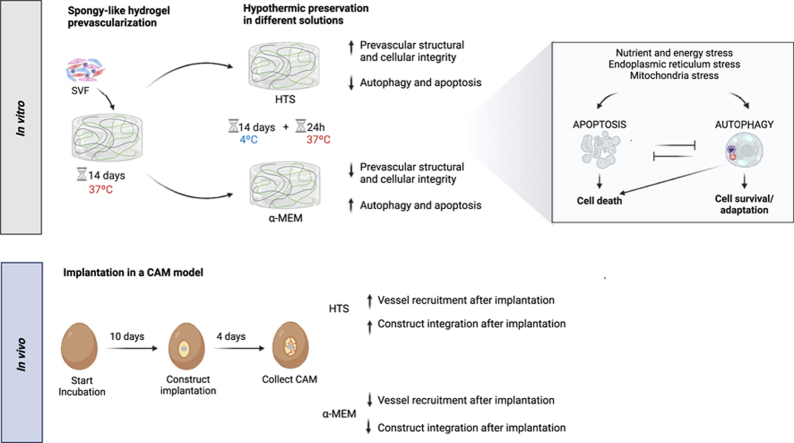

## 1 Introduction

The scarcity of human tissues and organs for transplantation poses a major challenge for healthcare systems worldwide, as the prevalence of chronic diseases and other severe medical conditions continue to rise, resulting in significant healthcare costs as well as in increased patient suffering and deaths [1,2]. Tissue engineering (TE) offer promising solutions to solve this issue. Through the use of bioartificial 3D matrices, composed of diverse materials, cells can be grown and matured into tissue-like constructs. Crucial to the viability of 3D thick constructs is the formation of a prevascular network, which enables the proper delivery of oxygen and nutrients to all cells, facilitating seamless integration with the surrounding tissue and overall regeneration. However, neovascularization from the host alone is often insufficient to ensure the viability of such constructs, and a prevascularization step that creates a capillary-like network before implantation, is necessary. The stromal vascular fraction (SVF) of adipose tissue has been suggested as a tool to achieve such prevascularization, as it contains various cell populations with intrinsic angiogenic potential [3]. In fact, cells present in this fraction have been shown to spontaneously assemble into prevascular networks in vitro without the need for angiogenic growth factor supplementation [[4], [5], [6]]. The establishment of prevascular networks in vitro using SVF has proven to enhance the engraftment of 3D tissue engineered constructs in vivo, by accelerating the vascularization process and enhancing the ability of the constructs to connect with the host's vascular networks [6]. But prevascularization is not the only challenge in TE. Creating tissue engineered constructs is a lengthy process that involves isolating and expanding cells, fabricating the constructs, and allowing them to mature in specialized facilities before they are ready for implantation. Preserving the properties of these constructs during transportation is a critical challenge to their widespread clinical use. The need to overcome this obstacle has been acknowledged by the field and effort has been placed on the development of advanced storage protocols that could extend the shelf life of the created construct, reducing waiting times for patients and logistical challenges [7,8]. Such advancements could also reduce costs, further promoting the adoption of tissue engineered products in clinical settings. However, it is challenging to preserve the viability of cells in complex systems like tissues or tissue engineered constructs, while maintaining the supportive matrix's structural properties. While cryogenic preservation is widely used for single cell preservation, it remains limited for complex systems like tissue engineered constructs. On the other hand, short-term preservation at hypothermic temperatures may be optimized for transportation while ensuring safety, quality, and functionality before administering the product to the patient. Hypothermia has in fact been tested as a potential short-term storage method for TE products [[9], [10], [11]]. Compared to cryopreservation, the milder temperatures of hypothermia can prevent cellular and structural damage caused by ice nucleation and osmotic stress from cryoprotectants, while slowing down biological processes and energy consumption in cells [12,13]. Although several hypothermic preservation solutions are currently available, most are focused on organ preservation. Hypothermosol (HTS) is a hypothermic preservation solution that has been specifically developed for TE products, inhibiting post-storage necrosis and apoptosis [14]. In fact, HTS has been demonstrated to significantly preserve the metabolic activity and extracellular matrix (ECM) integrity of cell sheet-like confluent human adipose stromal cells (hASCs) cultures during hypothermic storage for up to 7 days [11].

Considering the demonstrated potential of HTS for the hypothermic preservation of TE products, the objective of this work was to assess its efficiency on the preservation of capillary-like structures within prevascularized tissue engineered constructs. To accomplish this, a previously described gellan gum-based scaffold formulation [6] was prevascularized by taking advantage of SVF angiogenic capabilities. After preservation at 4 °C for 14 days using HTS or the basal media α-MEM, the integrity of the created prevascular networks vis-à-vis constructs before preservation was evaluated. *In vivo* functionality of the prevascularized 3D contructs was then assessed by examining blood vessel recruitment and integration with the host tissues after implantation in a chick chorioallantoic membrane (CAM) model.

## 2 Materials and methods

### 2.1 Gellan gum-based spongy-like hydrogels fabrication

Gellan gum/gellan gum divinyl sulfone - Arginylglycylaspartic acid (GG/GGDVS-RGD) spongy-like hydrogels were prepared as previously described [6,15]. Firstly, a modification of gellan gum (GG) with divinyl sulfone (DVS) [15] and post-functionalization with Arginylglycylaspartic acid (RGD) peptide was performed [6]. Briefly, Gelzan powder (0.25% w/v, Sigma, USA) was dissolved in deionized water, under stirring at 90 °C. After dissolution, DVS (Sigma, USA) was added to the GG solution and left to react for 1 h under stirring. After a purification step, (gellan gum divinyl sulfone) GGDVS was dialyzed against deionized water for 3 days at 37 °C and freeze-dried. For Thiol-cyclo-RGD peptide (RGD, Cyclo (-RGDfC), >95 % purity, GeneCust Europe) functionalization, an 800 μM solution of peptide was added to 0.25 % (w/v) GGDVS solution and left to react for 1 h at RT under agitation. Finally for the fabrication of spongy like hydrogels, a 0.25 % (w/v) GGDVS-RGD and a 0.5 % (w/v) GG solution were prepared. Hydrogels were prepared by mixing the GGDVS-RGD solution with the GG solution and cast into desired molds. Hydrogels were frozen at −80 °C overnight and then freeze-dried (Telstar, Spain) for 24 h to obtain GG/GGDVS-RGD dried polymeric networks. Spongy-like hydrogels were formed after rehydration of the dried polymeric networks with α-MEM.

### 2.2 Raman spectroscopy

To verify the presence of the RDG peptide in GG/GGDVS-RGD dried polymeric networks, raman spectroscopy was used. Spectra were collected using a confocal Raman microscope (LabRam Soleil from Horiba Scientific), equipped with a green laser (λex = 532 nm) with a maximum output of 75 mW. Spectra were collected using a 50x objective, a laser power of 61 mW (80%), and 1s of acquisition time with 10 accumulations, in the range of 300–1200 cm^−1^.

### 2.3 Micro-computed tomography (μ-CT)

The microarchitecture of dried polymeric networks was assessed using a high-resolution X-Ray Microtomography Skyscan 1072 scanner (Skyscan, Belgium). Samples were scanned in high-resolution mode with a pixel size of 11.31 μm (magnification of 23.30 × ) and an integration time of 1.7 s. The X-ray source operated at 35 keV energy and 215 μA current. Representative datasets consisting of 150 slices were converted into binary images using a dynamic threshold of 45e225 (gray values) to distinguish polymer material from pore voids. Three-dimensional reconstructions of selected regions within the bulk of the materials were generated, visualized, and registered using image processing software (CT-vox, SkyScan).

### 2.4 Scanning electron microscopy

Scanning electron microscopy (SEM) was used to analyze the microstructure of the dried polymeric networks. Prior to analysis, samples were sputter coated with a mixture of gold–palladium. A JSM-6010LV (JEOL, Akishima, Japan) microscope, operating with an accelerating voltage of 15 kV was used to capture images.

### 2.5 Water uptake and water content

To determine the water content of spongy-like hydrogels (5 mm diameter, 5 mm height), the weight of dried polymeric networks before (Wd) and after (Ww) immersion for 76 h at 37 °C in a phosphate buffered saline (PBS) solution was measured. The water uptake profile and water content were determined using the same principle, but samples were weighed at different time points over 7 days. Water content/uptake were calculated using the following equation:

Water Uptake/Content (%) = (Ww – Wd) / Wd x 100

### 2.6 Spongy-like hydrogels microarchitecture

Spongy-like hydrogels were stained with a highly concentrated (0.2 mg/mL) solution of the fluorescent dye 4′,6-diamidino-2-phenylindole (DAPI) (Biotium, USA). Images of fluorescently stained GG-based spongy-like hydrogels were acquired using a Zeiss LSM980 with Airyscan 2 Confocal Microscope (ZEISS, Germany).

### 2.7 Compressive modulus

The mechanical properties of spongy-like hydrogels (10 mm diameter, 5 mm height) were measured using a Instron 5543 mechanical testing device (Instron International Ltd., USA). After immersion in PBS for 24 h at room temperature, spongy-like hydrogels were placed on the device's base platform, compressed at a rate of 1 mm/s using a 50 N load cell, and the ratio of compressive stress to strain was recorded. Young's Modulus was calculated within the log10 linear region (0–1 % compressive strain).

### 2.8 Isolation of stromal vascular fraction cells

Human subcutaneous adipose tissue was obtained from surgical procedures performed at local hospitals, after patient's written informed consent, and in the scope of a collaboration protocol approved by the ethical committees of both institutions for this work (217/19; CEICVS 008/2019). Stromal vascular fraction was obtained as previously described [16,17]. Briefly, lipoaspirates were digested with a solution of 0.05% (w/v) collagenase type II (Sigma Aldrich, USA), for 45 min at 37 °C under agitation, and then centrifuged to obtain the SVF. The obtained SVF was incubated with red blood lysis buffer, centrifuged and the supernatant resuspended in α-MEM (Life Technologies, United Kingdom) supplemented with 10% fetal bovine serum (FBS) and 1% antibiotic/antimycotic. Finally, cell nuclei were stained using a solution of 3% (v/v) acetic acid (VWR, UK) and 0.05 wt % methylene blue (Sigma Aldrich, St. Louis, USA) in water to count nucleated cells.

### 2.9 Cell seeding

SVF cells were seeded on spongy-like hydrogels as previously described [6]. Briefly, a 30 μl cell suspension, comprising 1.5 × 10^6^ cells, was dispensed dropwise on top of the dried polymeric networks. Constructs were incubated for 30 min, at 37 °C, 5% CO2 to allow maximum cell entrapment within the structures and then fresh medium was added to make a total volume of 1 ml. Constructs were cultured for 14 days in α-MEM to allow the formation of a prevascular network.

### 2.10 Hypothermic preservation

In order to understand the impact of preservation at hypothermic temperatures, cell-laden constructs were stored in HTS (BioLife Solutions, USA) (100 mM Na^+^, 42,5 mM K^+^, 0,05 mM Ca^+^, 5 mM Mg^2+^, 17,1 mM Cl^−^, 10 Mm H_2_PO_4_^−^, 5 mM HCO_3_^−^, 25 mM HEPES, 100 mM Lactobionate, 20 mM Sucrose, 20 mM Mannitol, 5 mM Glucose, 6% Dextran-40, 2 mM Adenosine, 3 mM Glutathione, Trolox) or α-MEM, at 4 °C for 14 days. Controls comprising cell-laden constructs collected before preservation (BP) were also prepared. After storage, the preservation solutions were replaced with warm α-MEM, and cells allowed to recover for 24 h at 37 °C.

### 2.11 Angiogenesis proteome screening

In order to analyze the expression profile of angiogenesis-related proteins during the prevascularization process, conditioned media was collected and centrifuged to remove cell debris after 5, 10 and 14 days of culture. A control with basal media was also set up. Secreted proteins were analyzed using a Proteome Profiler human angiogenesis array (R&D Systems, USA) in accordance with manufacturer guidelines. Briefly, conditioned media were incubated with an assay-specific detection antibody cocktail for 1 h at room temperature. After a membrane blocking step, samples containing the antibody cocktail were added to the respective membrane and incubated overnight at 4 °C under gentle shaking. Membranes were then washed with 1 × wash buffer, incubated with streptavidin-HRP for 30 min, and washed again. Membranes were incubated with Chemi Reagent Mix for 1 min, and spots were detected using chemiluminescence in an Odyssey Fc Imaging System (LI-COR, USA) and quantified by densitometry using Image studio 5.2 software (LI-COR, USA).

### 2.12 Immunocytochemistry

Constructs were fixed in a buffered formalin solution. Non-specific binding was blocked with a 3% (w/v) bovine serum albumin (BSA) solution and constructs were then incubated overnight at 4 °C with primary antibody mouse anti-human CD31 (1:30) (Dako, United Kingdom). After repeated washes in PBS, secondary antibody Alexa Fluor 594 donkey anti-mouse (1:500) (Molecular probes, USA) was incubated with cells for 4 h at RT. Cells were washed in PBS and cell nuclei was counterstained with DAPI. Constructs were then analyzed in a confocal laser scanning microscope Zeiss LSM980 with Airyscan 2 (ZEISS, Germany).

### 2.13 Prevascular network characterization

Prevascular networks stained for CD31 were quantitatively analyzed using angiogenesis ImageJ 1.54b (National Institutes of Health, Bethesda, MD, USA). The number of nodes, junctions, meshes, segments, branches and isolated segments were quantified blindly in 9 random representative images for each condition (Fig. S1).

### 2.14 Viability staining

Cell-laden spongy-like hydrogels were incubated with calcein-AM (Ca-AM, 1 μg mL^−1^, Invitrogen, USA), propidium iodide (PI, 2 μg mL^−1^, Invitrogen, USA) and Hoechst (20 mM, Thermo Fisher Scientific, USA) for 1 h at 37 °C. Cell viability was observed with a confocal laser scanning microscope (SP8, Leica Microsystems CMS GmbH). In the analysis, cells stained with calcein-AM were categorized as live cells, while cells stained with PI were classified as dead cells. To determine the counts of live and dead cells for each condition, we conducted quantification across nine randomly selected images using the Cell Counter plugin within ImageJ 1.54b (National Institutes of Health, Bethesda, MD, USA). The percentage of live cells was calculated according to the following formula:

% live cells = number of live cells/total number of cells × 100

### 2.15 DNA quantification

Spongy-like hydrogels were incubated with 0.5 mL of ultra-pure water for 1 h at 37 °C and then frozen at −80 °C for 24 h. Afterwards, materials were heated to 70 °C for 30 min, macerated and dissolved with the help of a vortex and centrifuged for 5 min at 1000*g*. The supernatant with the DNA content was collected. An accuclear ultra high sensitivity dsDNA quantitation kit (Biotium, USA) was used for double strand DNA (dsDNA) quantification. Samples were prepared according to manufacturer instructions and fluorescence was read in fluorescence spectrometer (Jasco FP-8500, Japan).

### 2.16 Inflammatory and angiogenic proteome profiler

Cell culture supernatants were analyzed using a human cytokine magnetic panel (IL-8, IL-18, IL-2, IL-10, IL-1β, IL-6, VEGF, PDGF-BB, HGF, FGF-b, Ang-2, PAI-1, PIGF, Ang-1, MCP-1) (R&D Systems, USA) according to the manufacturer's instructions. Briefly, samples were centrifuged (16,000g 4 min), collected to new vials and prepared to be used at an appropriated dilution. After adding 50 μL of each sample/standard plus 50 μL of microparticle cocktail per well, the prepared plate was incubated for 2 h at RT on a horizontal orbital microplate shaker (VWR, US) set at 800 rpm. Following 3 washing steps, 50 μL of biotinylated antibody was added per well and the plate was incubated for 1 h at RT on a horizontal orbital microplate shaker (VWR, US) set at 800 rpm. After incubation, samples were washed and then incubated with 50 μL of streptavidin-PE for 30 min at RT on the horizontal orbital microplate shaker (VWR, US) set at 800 rpm. Samples were washed and then read in a Luminex MAGPIX Instrument System (Thermo Fisher Scientific, USA). Biological triplicates of each experiment (n = 3) were measured in duplicate. The concentration of each analyte was calculated using the Luminex xPONENT 4.2 software (Thermo Fisher Scientific, USA).

### 2.17 Apoptosis assay

Prevascularized spongy-like hydrogels before and after preservation were assessed for apoptosis. As a positive control for apoptosis, prevascularized spongy-like hydrogels were incubated for 24 h in α-MEM supplemented with 10% FBS, 1% Antibiotic/Antimicotic and 100 μg mL^−1^ of mitomycin to induce apoptosis. After incubation, cells were lysed, and immunoblotting assays were performed for caspase 3 apoptosis-related protein.

### 2.18 Autophagy flux analysis

Prevascularized spongy-like hydrogels before and after preservation were incubated with or without 10 μM of metformin (Sigma-Aldrich, USA), for 4 h. Furthermore, in order to block the autophagy flux and allow the accumulation of LC3-II, 2 h before the end of the treatment, in each condition, cells were also incubated with 10 nM of bafilomycin (Baf A1) (Sigma-Aldrich, USA). After incubation, cells were lysed, and immunoblotting assays were performed for autophagy-related proteins. To quantify autophagy synthesis, the ratio of the densitometric values of the cells treated with metformin and Baf A1 against those for condition without metformin but with Baf A1 treatment was determined. To quantify autophagy degradation, the ratio between the densitometric values of cells treated with metformin in the presence or absence of Baf A1 was determined, according to autophagy standard guidelines. Autophagy flux was assessed by comparing relative LC3-II levels under autophagy induction with metformin against basal conditions for each preservation condition or BP. A positive flux denotes autophagy induction, while a negative flux indicates a blockage in the process [18].

### 2.19 Western blot

For protein extraction, 50 μl of laemmli buffer (0.05% (w/v) bromophenol blue, glycerol (30% (v/v)), 5 M EDTA, NaOH solution, 6% (w/v) SDS and 1.875 M Tris pH 8.8 solution) and 1% (v/v) Dithiothreitol, (all from Sigma-Aldrich, USA) were used. Samples were macerated and denatured for 20 min at 45 °C followed by 20 min 65 °C and finally 10 min at 90 °C. For the immunoblotting assay, 10 μg of total protein extract were resolved in a 12% SDS gel and transferred to a nitrocellulose membrane for 10 min in Trans-Blot Turbo transfer system (Bio-Rad, USA). Membranes were blocked in Tris-buffered saline (TBS) with 0.1% tween 20 (TBS-T) containing 5% BSA and afterwards incubated overnight at 4 °C, with the polyclonal primary antibodies in 1% BSA: rabbit anti-LC3A/B Antibody (1:1000) (Cell-Signaling Technology, USA), mouse anti‐p62 (1:1000) (Abcam, UK), rabbit anti-human caspase 3 (1:700) (Cell Signaling Technology, USA) and mouse anti‐alpha‐actin (Millipore, USA). Secondary antibodies (HRP, anti‐rabbit, anti‐mouse) were from Bio‐Rad, USA (1:5000). Blots were treated with the SuperSignal West Femto Maximum Sensitivity Substrate (Thermo Fisher Scientific, USA) or Clarity Western ECL Substrate (Bio‐Rad, USA). Digital images and densitometry analysis of the bands were obtained in a ChemiDoc XRS System (Bio‐Rad, USA) with Quantity One software V4.6.5 (Bio‐Rad, USA), respectively.

### 2.20 In ovo implantation

A CAM assay was performed as previously described [19]. White fertilized chicken eggs were incubated at 37 °C in a temperature incubator (Termaks KB8000, Norway) for 3 days. After this, a window was opened into the shell to evaluate embryo viability. Prevascularized spongy-like hydrogels BP (*n* = 10), and after preservation with HTS (*n* = 10) or α-MEM (*n* = 10) were implanted on the CAM at day 10 of embryonic development, and the eggs returned to the incubator at 37 °C. Control groups with empty materials (*n* = 10) and without the material (*n* = 10) were also set up. After 4 days of implantation, embryos were sacrificed with 4 % (*v/v*) paraformaldehyde and subsequent incubation at −80 °C for 10 min. Then, the implanted materials with adjacent portions of CAM were cut and fixed with 4% (*v/v*) paraformaldehyde. Ex ovo images were captured using a Stemi 2000-C stereo microscope (ZEISS, Germany).

### 2.21 New vessel quantification

The obtained ex ovo images were processed using ImageJ 1.54b (National Institutes of Health, Bethesda, MD, USA). Images were cropped to a defined area of 1000 × 1000 pixels, considering the implanted construct in the center of the image. The vascular density (area%) and vessel dimeter (mm) were quantified using the Vessel Analysis plugin included in ImageJ 1.54b (National Institutes of Health, USA).

### 2.22 Histological analysis

After formalin fixation, CAM explants were processed in a MICRON STP120-2 spin tissue processor (MICRON, Germany), embedded in paraffin (Thermo Scientific, USA), and serially sectioned into 4 μm-thick sections.

### 2.23 Hematoxylin and eosin staining

Hematoxylin and eosin (H&E) staining was performed in CAM sections. Briefly, sections were deparaffinized with xylene, rehydrated in graded ethanol series and stained with hematoxylin and eosin in an MICROM HMS740 automatic stainer (MICROM, Germany). Afterwards, sections were dehydrated and mounted with resinous mounting medium Entellan® (Merck, Germany). Histological sections were analyzed under a Leica DM750 microscope (Leica, Germany).

### 2.24 Membrane-scaffold ration

H&E images were processed using ImageJ 1.54b (National Institutes of Health, Bethesda, MD, USA). To do so, a region of interest, with a width of 900 pixels and a height determined by the construct, was defined for each image. After defining the area of non-integrated “empty” scaffold in the image, the corresponding region was measured. The same procedure was performed for the CAM membrane area (Fig. S6). Finally, the membrane-scaffold ratio was obtained using the following formula:

Membrane-scaffold ratio = CAM membrane area / Empty scaffold area

### 2.25 Immunohistochemistry

CAM sections were deparaffinized and rehydrated in an MICROM HMS740 automatic stainer (MICROM, Germany). Immunohistochemical analysis was performed using a streptavidin–biotin peroxidase complex system. Briefly, after rehydration, slides were subjected to heat-induced antigen retrieval with 10 mM citrate buffer at pH = 6 for 2 min at 98 °C. The slides were washed with PBS and then incubated with 3% hydrogen peroxide for 10 min to inactivate endogenous peroxidases. Another washing step was performed, and nonspecific binding was blocked with a 2.5% (*v/v*) horse serum (Vector Labs, USA) for 30 min, before overnight incubation at 4 °C with mouse anti-human CD31 (1:30) (Dako, UK). Sections were washed with 0.1% tween in PBS and incubated with a secondary biotinylated antibody (Vector Labs, USA) for 20 min. After thoroughly washed with 0.1% tween in PBS, samples were incubated with streptavidin-HRP (Vector Labs, USA) for 20 min, followed by 3,3′diaminobenzidine (DAB) incubation (Vector Labs, USA). Finally, all sections were counterstained with Mayer's hematoxylin, dehydrated and mounted with resinous mounting medium Entellan® (Merck, Germany).

Human CD31 stained area quantification was performed using ImageJ 1.54b (National Institutes of Health, Bethesda, MD, USA) as previously described [20]. Shortly, images were split in to their respective RGB channels. A manual threshold was applied to each experimental replicate, and the percentage of area with signal above the threshold was determined. Positive stained area was calculated across 4 randomly selected fields of at least 4 different sample section, acquired using a 40x objective.

### 2.26 *In situ* hybridization

The presence of human cells within the implantation area was assessed using a human-specific DNA probe, according to the respective detection system BIO-AP REMBRANDT® Universal DISH & Detection kit (PanPath, The Netherlands). Briefly, after deparaffinization, proteolytic digestion was performed using a pepsin-HCL solution for 30 min at 37 °C, followed by dehydration in graded ethanol series. Sections were air-dried, and 1 drop of the probe was applied and covered with a coverslip. Samples were incubated at 95 °C for 5 min for DNA denaturation and then for 16 h at 37 °C in a moisturized environment for hybridization to occur. Samples were then washed in Tris-buffered saline (TBS) and incubated for 10 min with the stringency wash buffer. After rinsing with TBS, the detection was performed, and color was permitted to develop for 5 min at 37 °C in the dark. Samples were washed with water, counterstained with nuclear fast red, and observed under a Leica DM750 microscope (Leica, Germany).

The number of human cells was performed using ImageJ 1.54b (National Institutes of Health, Bethesda, MD, USA). Positive stained cells were manually counted across 3 randomly selected fields of at least 3 different sample section, acquired using a 40x objective.

## 3 Results

**Fig. 1:**
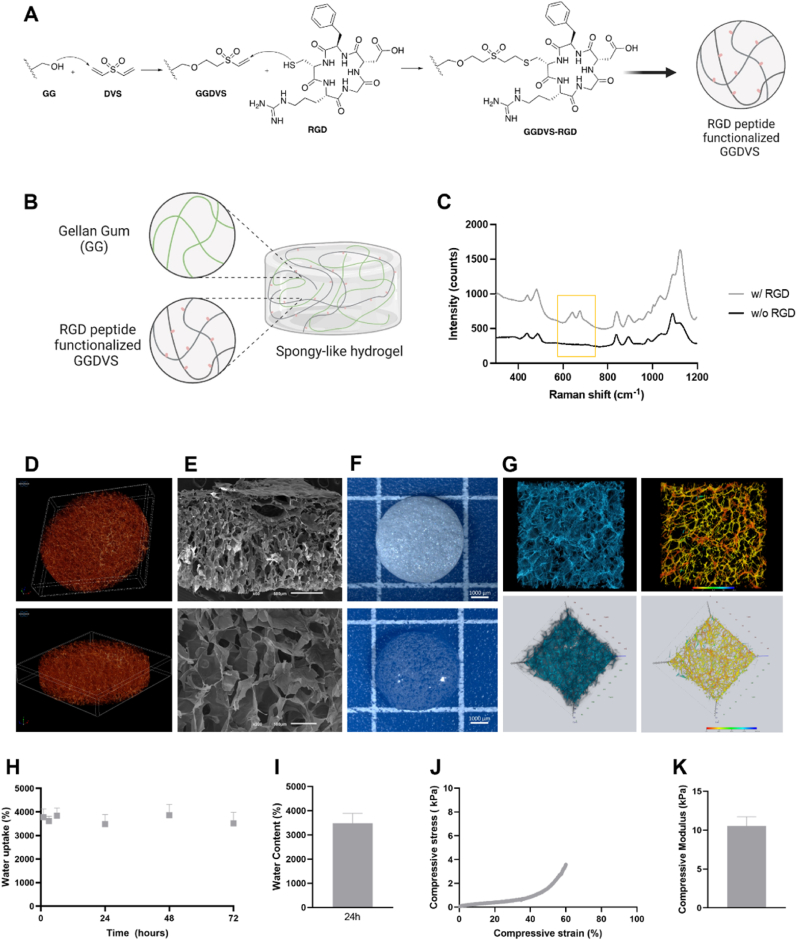
**RGD functionalized spongy-like hydrogels characteristics**. **(A)** Schematic representation of the chemical modification of GG with DVS and subsequent functionalization with RGD. **(B)** Schematic representation of the production of GGDVS-RGD spongy-like hydrogels. **C)** Raman spectra of GG, with and without RGD, acquired within the spectral range of 300–1200 cm^−1^**(D)** 3D reconstruction of a representative region in the bulk of dried polymeric networks after μ-CT acquisition. **(E)** Representative scanning electron microscopy images of the dried polymeric networks. **(F)** Representative macroscopic images of dried polymeric networks before (top) and after (bottom) hydration. Scale bar: 1000 μm. **(G)** 3D reconstruction of representative regions in the bulk of spongy-like hydrogels after confocal microscopy acquisition. The structures are color-coded based on their proximity to the top, with red indicating closer proximity and blue indicating greater distance (right). **(H)** Water uptake of dried polymeric networks after hydration with PBS, at 37 °C, for 72h. Results are expressed as mean ± stddev. **(I)** Water content of spongy-like hydrogels after hydration with PBS, at 37 °C, for 24h. Results are expressed as mean ± stddev. **(J)** Representative curve of compressive stress (kPa) vs compressive strain (%). **(K)** Compressive Modulus of spongy-like hydrogels (n = 8). Results are expressed as mean ± stddev.

**RGD functionalized spongy-like hydrogels characteristics.** After functionalizing GGDVS with RGD peptide (Fig. 1A), the dried polymeric networks were produced by combining GGDVS-RGD with unmodified GG (Fig. 1B) [6,15]. The presence of RGD peptide was confirmed by Raman spectroscopy (Fig. 1C). Scanning electron microscopy and μ-CT revealed that the dried polymeric networks had a wide microarchitecture with a semi-interpenetrating network structure (Fig. 1D and E). After hydration, the dried polymeric networks formed spongy-like hydrogels (Fig. 1F). Upon immersion in aqueous solutions such as PBS, these dried structures exhibited rapid water absorption, reaching a saturation point just 1 h after immersion at 3779.9 ± 351.0% (Fig. 1H). This plateau was sustained for up to 72 h. Importantly, the microarchitecture and interconnected structure previously observed in the dried polymeric networks remained intact after saline solution hydration (Fig. 1G). After reaching the equilibrium for water uptake (24h) (Fig. 1I), we proceeded to assess the mechanical properties of the GGDVS-RGD spongy-like hydrogels. These hydrogels demonstrated the ability to compress up to 80% of their height without fracturing (Fig. 1J), showcasing a compressive modulus of 10.52 ± 1.209 kPa (Fig. 1K).

### 3.1 Spongy-like hydrogels support the SVF organization and formation of a prevascular network in the absence of extrinsic angiogenic growth factors

**Fig. 2:**
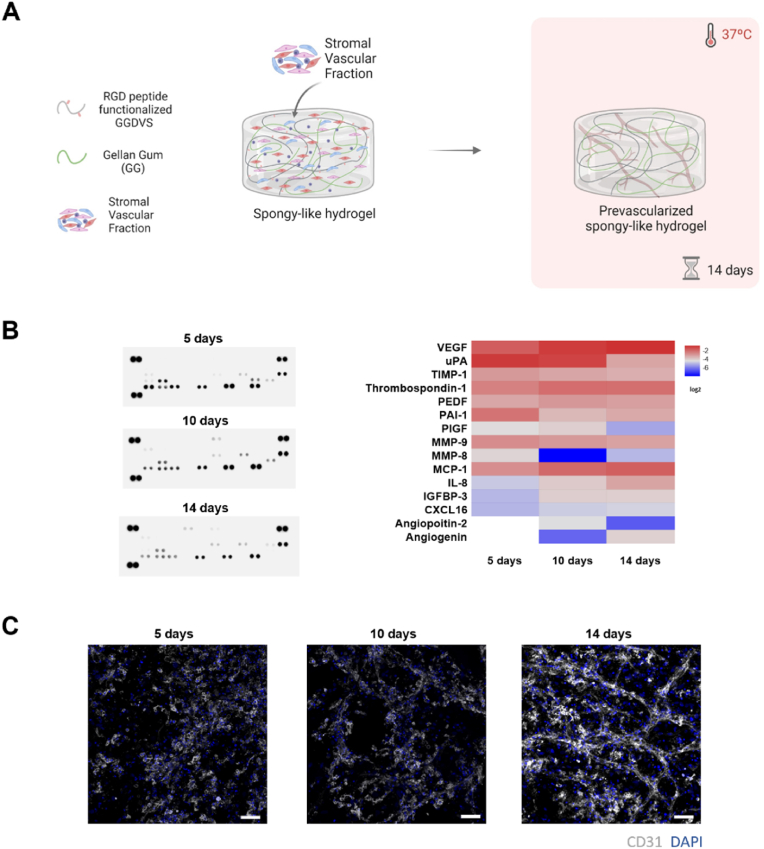
**Spongy-like hydrogels support the SVF organization and formation of a prevascular network in the absence of extrinsic angiogenic growth factors**. **(A)** Schematic representation of the rationale of using GGDVS functionalized with RGD peptides to capture cells involved in vasculogenesis from the stromal vascular fraction (SVF) of adipose tissue. **(B)** Angiogenic profile of prevascularized GGDVS-RGD spongy-like hydrogels secretome at different culture times. Conditioned media was collected on days 5, 10 and 14 for dot blot analysis of angiogenesis-related factors. Protein expression profiles were measured using mean integrated intensity and normalized to the reference spots. **(C)** Representative immunocytochemistry images of the organization of SVF-derived CD31 (white) expressing cells within GGDVS-RGD spongy-like hydrogels after 5, 10 and 14 days of culture in basal media. Cell nuclei were counterstained with DAPI (blue). Scale bar: 50 μm.

To create a prevascular network in the produced constructs, SVF was isolated from adipose tissue, seeded onto the hydrogels and cultured for 14 days, with media changes after 5 and 10 days of culture (Fig. 2A) [6]. To ensure the angiogenic potential of the freshly isolated SVF cells, tube formation was evaluated under 2D conditions on standard TCPS (Fig. S2). Since angiogenic and vasculogenic signaling is crucial in vasculature formation, the secretion of specific proteins by SVF cells at 5, 10 and 14 days of culture was assessed. The levels of angiogenic/vasculogenic-related proteins changed over time for most of the factors analyzed (Fig. 2B). Factors involved in ECM remodeling, namely uPA, PAI-1, TIMP-1, MMP-9, and MMP-8 showed a decrease in levels over time, while MCP-1, IL-8, thrombospondin-1, and VEGF increased over the first 10 days of culture and remained constant thereafter (Fig. 2B). At early time points, endothelial CD31-positive cells displayed a non-organized pattern as demonstrated by immunocytochemistry (Fig. 2C). As levels of angiogenic/vasculogenic proteins increased and stabilized after 10 days of culture, the formation of a prevascular network became apparent, culminating in the development of a complex and interconnected network by the 14th day.

### 3.2 Hypothermosol preserved prevascular networks after 14 days of hypothermic preservation

**Fig. 3:**
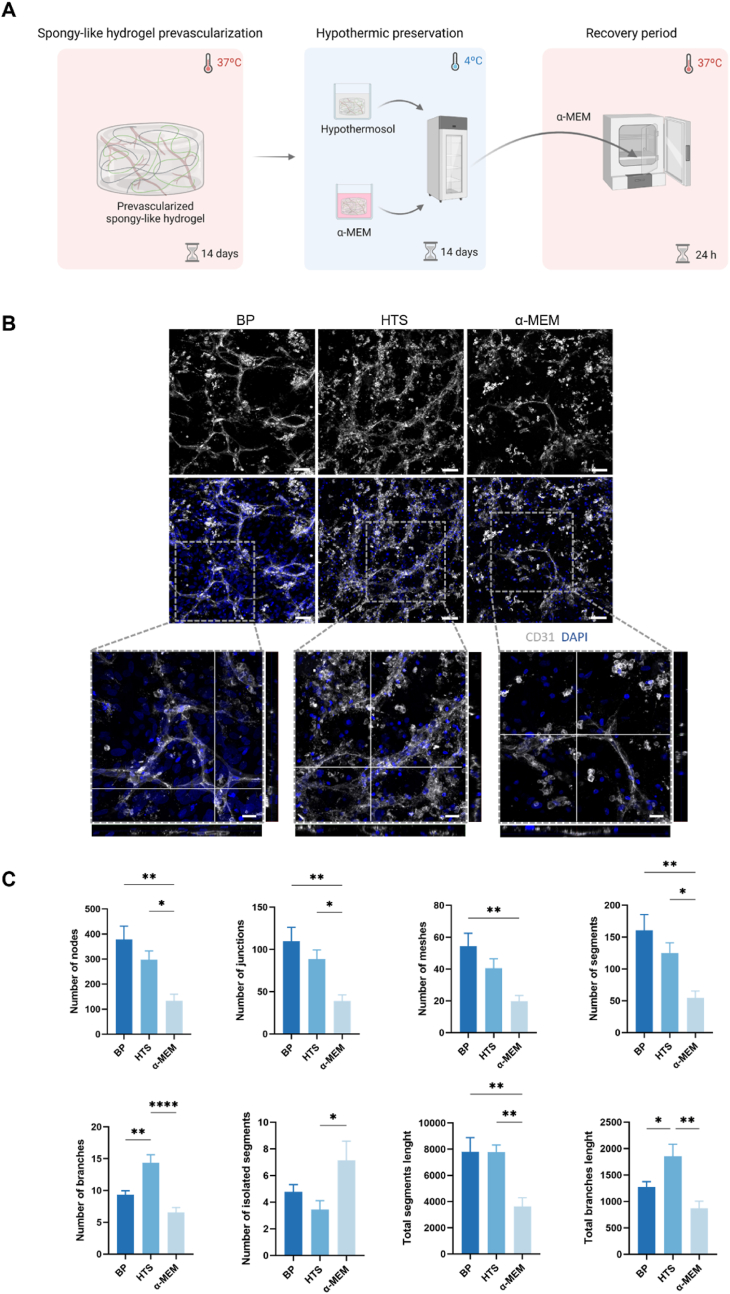
**Hypothermosol preserved the created prevascular network after 14 days of hypothermic preservation**. **(A)** Schematic representation of the preservation methodology used. **(B)** Representative immunocytochemistry images of the organization of SVF-derived CD31 (white) expressing cells within GGDVS-RGD spongy-like hydrogels before preservation (BP) and after 14 days of preservation with HTS and α-MEM at 4 °C + 24h recovery at 37 °C. Detail of the capillary-like structures formed showing several lumens. Cell nuclei were counterstained with DAPI (blue). Scale bar: 50 μm (top) and 20 μm (bottom). **(C)** Quantification of the number of nodes, junctions, meshes, segments, branches, isolated segments, segments length and branches length in the capillary-like structures formed after 14 days of culture and after 14 days of preservation with HTS and α-MEM at 4 °C + 24h recovery at 37 °C in basal media. Results are expressed as mean ± SEM and analyzed using one-way ANOVA with Tukey multiple comparison post-test (*p < 0.0332, **p < 0.0021, and ****p < 0.0001).

We then looked at the possibility of preserving the created prevascular network for a period that allows storage of the engineered constructs until its clinical application. The prevascularized constructs were stored at 4 °C using HTS or α-MEM as storage solutions for 14 days. After this period, storage solutions were replaced by culture media and the construct allowed to recover for 24h under physiological conditions (Fig. 3A).

When α-MEM was used as the storage solution, a visible reduction in the extension and complexity of the prevascular network was observed, along with a decrease in cellular content (Fig. 3B). The number of nodes and segments showed a significant decrease, while the number of isolated segments significantly increased, indicating a less complex structure after preservation with α-MEM (Fig. 3C). In contrast, when HTS was used as a storage solution, the complexity and interconnectivity of the created prevascular network was preserved in a similar state as BP, as evidenced by the expression pattern of CD31 (Fig. 3B). Analysis of the number of nodes, junctions, meshes, segments, and segment length showed unchanged values when preservation was performed with HTS (Fig. 3C). Interestingly, the number of branches and total branch length significantly increased after preservation with HTS (Fig. 3C). Furthermore, although the proportion of living cells did not significantly change after hypothermic preservation, regardless of the storage solution used, dsDNA analysis confirmed the effectiveness of HTS in preserving cellular content during the preservation period, in opposition to the α-MEM condition (Fig. S3). Furthermore, the use of calcein staining allowed the observation of important differences in cell morphology after preservation. Cells preserved with HTS maintained their spindle-shaped morphology, similar to BP, indicating their preserved structural integrity. On the other hand, cells preserved with α-MEM displayed a more rounded and fragmented morphology, suggesting cellular damage (Fig. S3).

### 3.3 Hypothermosol protects cells from oxidative stress while conserving SVF secretome profile

**Fig. 4:**
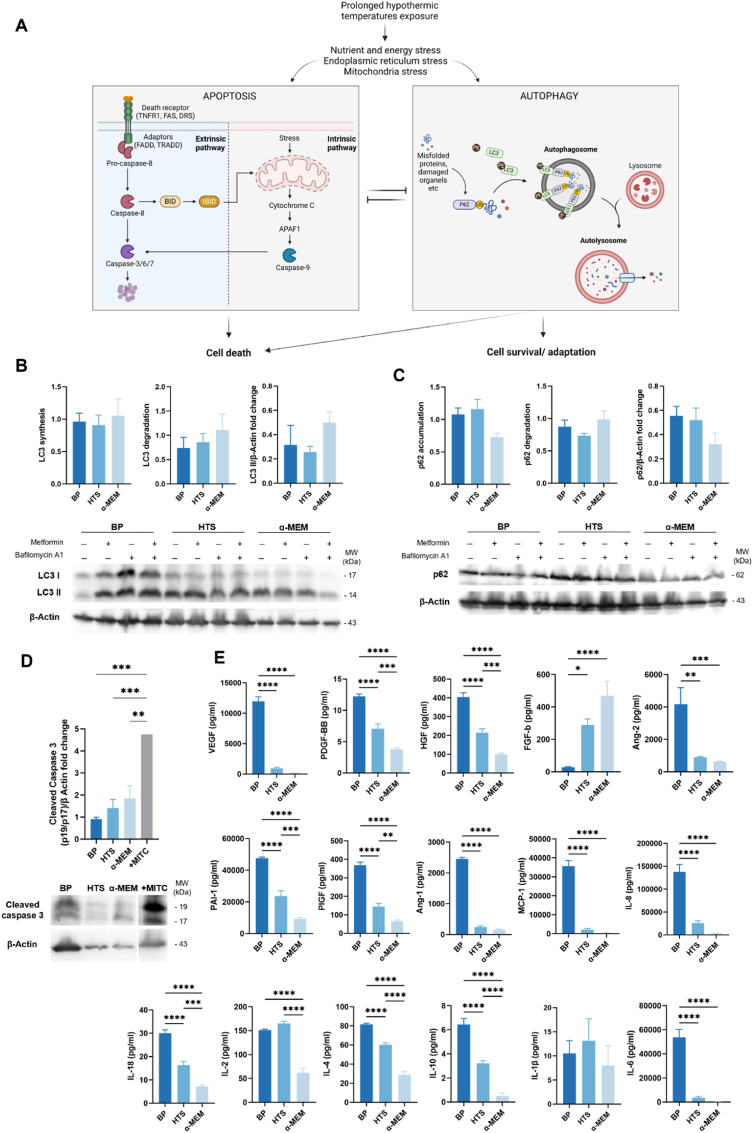
**Hypothermosol protects cells from oxidative stress conserving SVF population heterogenicity**. **(A)** Schematic representation of the hypothesized relationship between apoptosis and autophagy activation in response to hypothermic insult. (**B,C)** Autophagic flux in SVF cells before preservation (BP) and after 14 days of preservation with HTS and α-MEM at 4 °C + 4h recovery at 37 °C in basal media. **(B)** LC3 synthesis, degradation and protein level of LC3 with correspondent representative Western blot bands. **(C)** p62 synthesis, degradation and protein level of p62 with correspondent representative Western blot bands. **(D)** Protein level of cleaved caspase 3 in SVF cells before preservation (BP) and after 14 days of preservation with HTS and α-MEM at 4 °C + 4h recovery at 37 °C in basal media and after exposure or mitomycin C (+MITC) with correspondent representative Western blot bands. All plotted Western blot data was determined in relation to β-actin expression. **(E)** Angiogenic and inflammatory profile of prevascularized GGDVS spongy-like hydrogels secretome. Conditioned media was collected before preservation (BP) and after 14 days of preservation with HTS and α.-MEM at 4 °C + 24h recovery at 37 °C in basal media for multiplex analysis of angiogenesis and inflammation related factors. Results are expressed as mean ± SEM and analyzed using one-way ANOVA with Tukey multiple comparison post-test (*p < 0.0332, **p < 0.0021, and ****p < 0.0001).

After assessing the effects on cells morphology and organization, it was important to investigate the effects of hypothermia on cellular processes, specifically focusing on autophagy and apoptosis. Understanding the interplay between these cellular responses is a crucial first step for elucidating the mechanisms underlying the cellular adaptations during hypothermic conditions (Fig. 4A).

To investigate whether autophagy is affected by hypothermic temperature exposure we evaluated autophagic flux before and after hypothermic preservation using an immunoblot assay. Measurements were performed in both basal and stimulated conditions. For the latter, cells were treated with the autophagy inducer metformin during 4 h [21]. Autophagy flux was assessed by LC3 processing performed according to gold standard guidelines [22]. During autophagy, LC3-I is conjugated to phosphatidylethanolamine (PE) to form LC3-II, which co-localizes at the autophagosome membranes thus reflecting the number of autophagosomes and autophagy-related structures. LC3-II has a high recycling turnover, synthesis and degradation. Therefore, we used an autophagosome-lysosome fusion inhibitor, bafilomycin A1 (Baf A1), to block this process, allowing the accumulation of autophagosomes. Under normal autophagic flux an accumulation of LC3-II is expected, and alterations in LC3-II accumulation suggest an imbalance by defaulted synthesis and/or degradation. LC3-II synthesis was assessed by computing the ratio of the LC3-II protein levels from stimulated conditions (treated with metformin) plus Baf A1 over the LC3-II protein levels of the samples under basal conditions with Baf A1 alone. The LC3-II degradation was also assessed by computing the ratio of the LC3-II protein levels in stimulated conditions plus Baf A1 over the LC3-II protein levels obtained under stimulated conditions only [22]. Although the total levels of LC3-II did not show significant differences among the conditions, there was a noticeable increase of this protein in constructs preserved with α-MEM (Fig. 4B, Fig. S4A). In what regards LC3-II accumulation and degradation, we did not observe significant differences between the various conditions. Finaly, we evaluated autophagy flux by determining the relative levels of LC3-II in conditions of autophagy stimulation (metformin-treated), both with and without Baf A1. This assessment involved comparing these levels with the differences observed in LC3-II levels in the basal condition, both with and without Baf A1. The data revealed that storage in HTS resulted in a blockade of autophagy flux, as evidenced by a reduction in LC3-II levels in stimulated conditions. Conversely, using α-MEM as the storage solution, it was noticed a slight increase in LC3-II under stimulated conditions, suggesting at least the maintenance of autophagy flux (Fig. 4B, Fig. S4D).

Another autophagy substrate, p62, has been widely used as an indicator of autophagy activity [23]. Alterations in p62 accumulation and degradation also suggest changes in autophagic flux. Additionally, it is described that the turnover of p62 occurs in the same conditions as LC3. Generally, the activation of autophagy leads to a decrease in p62 protein levels. Although not achieving statistical significance, our results demonstrated a clear trend for the reduction of total p62 levels for constructs preserved with α-MEM (Fig. 4C, Fig. S4B). Furthermore, when analyzing p62 accumulation and degradation, we observed a decrease in p62 accumulation when α-MEM was used as the preservation solution, while there were no significant changes observed in p62 degradation.

To assess the activation of the apoptotic cascade, we analyzed the expression of caspase-3, an effector caspase (Fig. 4D, Fig. S4C). Control cells treated with mitomycin C, a known apoptosis inducer, exhibited the presence of two bands at approximately 19 kDa and 17 kDa, corresponding to the active forms of caspase-3. Similarly, in all experimental conditions, we observed the presence of these two active caspase-3 fragments. However, constructs preserved with -MEM showed a noticeable trend of increased caspase-3 activation compared to the other conditions.

Taking into account the observed decrease in vessel-like structures following preservation with α-MEM and considering prior research showcasing the vulnerability of specific cell types, particularly endothelial and immune cells [[24], [25], [26], [27]], to hypothermic temperatures, we aimed at gaining deeper insights into the status of these cells. We conducted an analysis of the secretome of prevascularized constructs both before and after preservation, focusing on angiogenic/vasculogenic and inflammatory cytokines (Fig. 4E). Among the angiogenic/vasculogenic-related factors, there was a notable decrease in the levels of VEGF, Ang-1, and Ang-2 following preservation. The use of α-MEM as a preservation medium led to a more significant reduction in the levels of PAI-1, PDGF-bb, HDF, and PIDF. Interestingly, there was an opposing trend observed in FGF expression, which increased after preservation, particularly when α-MEM was employed. As for inflammatory cytokines, a similar trend emerged, revealing a more pronounced decline in the levels of both anti-inflammatory and pro-inflammatory cytokines like MCP-1, IL-6, IL-18, IL-4, IL-10, IL-8, and IL-2 after preservation with the use of α-MEM. Interestingly, the levels of IL-1β remained unchanged in both conditions.

### 3.4 Preservation in HTS does not affect the in vivo angiogenic potential of prevascularized spongy-like hydrogels

**Fig. 5:**
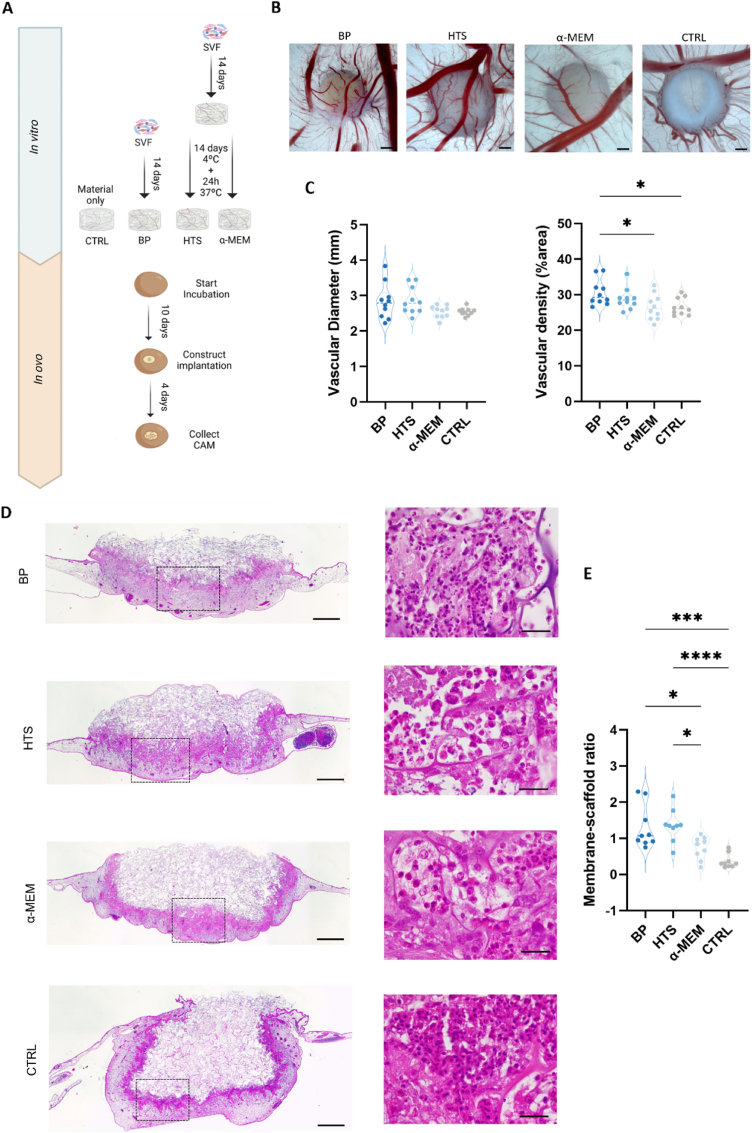
**Preserved prevascularized spongy-like hydrogels maintain their angiogenic potential**. **(A)** In vivo experimental design. **(B)** Representative micrographs, scale bar: 1000 μm, and **(C)** Quantification of recruited vessels after 4 days of implantation of prevascularized GGDVS-RGD spongy-like hydrogels and before preservation (BP) and after 14 days of preservation with HTS and α-MEM at 4 °C + 24h recovery at 37 °C in basal media. Data are presented as a violin plot illustrating the kernel density distribution frequency of recruited vessels and analyzed using one-way ANOVA with Tukey multiple comparison post-test (*p < 0.0332). **(D)** Representative micrographs of hematoxylin and eosin staining in prevascularized GGDVS-RGD spongy-like hydrogels before preservation (BP) and after 14 days of preservation with HTS and α-MEM at 4 °C + 24h recovery at 37 °C in basal media. Scale bar: 500 μm (left), 20 μm (right) **(E)** Quantification of membrane-scaffold ratio after 4 days of implantation of prevascularized GGDVS-RGD spongy-like hydrogels before preservation (BP) and after 14 days of preservation with HTS or α-MEM at 4 °C + 24h recovery at 37 °C in basal media. A region of interest, with a width of 900 pixels and a height determined by the construct, was defined for each image, followed by the calculation of the area corresponding to both the CAM membrane and the empty (without CAM invasion) scaffold. Data are presented as a violin plot illustrating the kernel density distribution frequency of stained area and analyzed using one-way ANOVA with Tukey multiple comparison post-test (*p < 0.0332, ***p < 0.0002, and ****p < 0.0001).

As stated above, the preservation of prevascular networks during hypothermic preservation is essential. Therefore, we aimed to determine whether prevascularized constructs subjected to hypothermic conditions maintained their in vivo angiogenic potential using the CAM model. Prevascularized spongy-like hydrogels were implanted for 4 days in the CAM of chicken eggs before and after preservation with HTS or α-MEM. A control group comprising spongy-like hydrogels without cells was also implanted (Fig. 5A). The area around the implantation site was fixed, photographed, and excised and paraffin-embedded for angiogenesis evaluation and quantification. Results revealed host vessel recruitment in all conditions (Fig. 5B). However, vascular density was significantly lower in constructs preserved with α-MEM and in the control group, indicating weaker vessel recruitment than BP (Fig. 5C). A trend towards lower vessel caliber was observed in spongy-like hydrogels preserved with α-MEM and control groups, but no statistically significant differences were found. Histological analysis with H&E staining revealed an immune response characterized by heterophil infiltration in all groups (Fig. 5D). Prevascularized spongy-like hydrogels, both before and after preservation, showed greater host tissue ingrowth towards the bulk of the constructs than the control group, indicating improved integration. Nevertheless, host tissue ingrowth was much less pronounced in hydrogels preserved with α-MEM and negligible for the control group (Fig. 5E, Fig. S5A and Fig. S6).

**Fig. 6:**
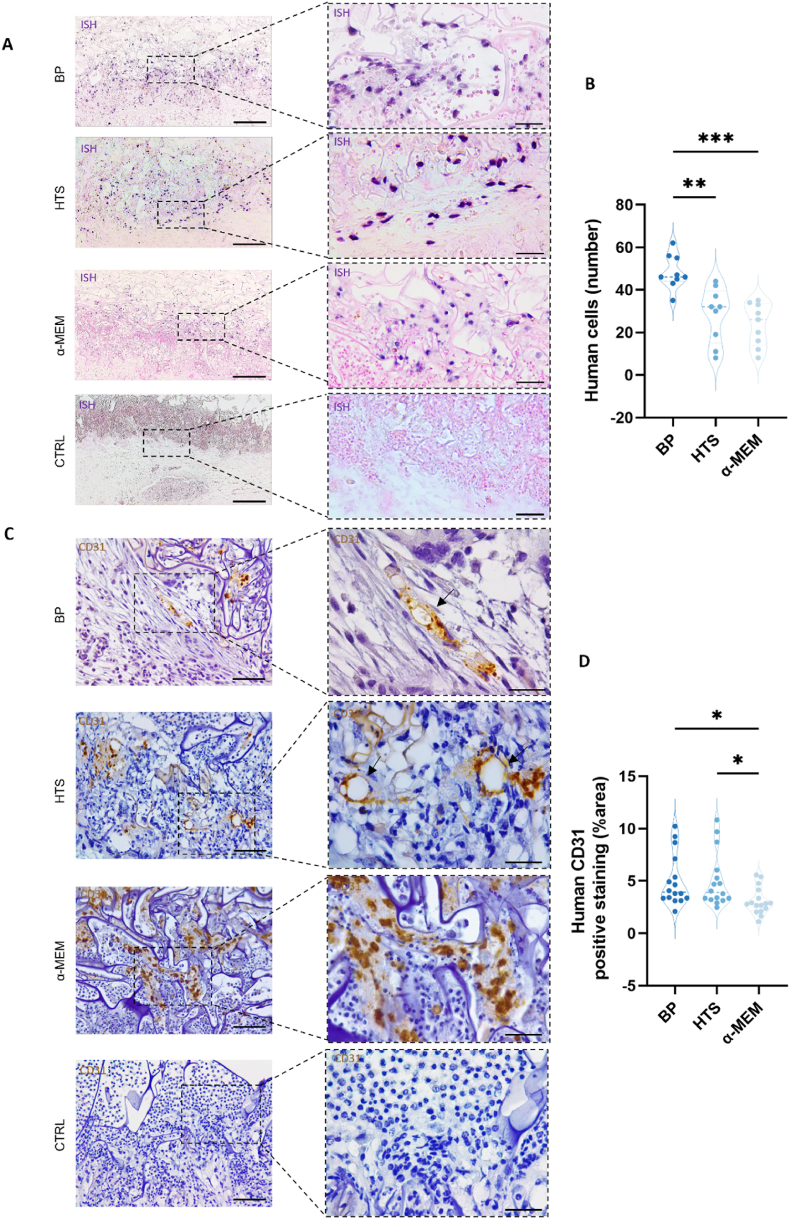
**Preserved prevascularized spongy-like hydrogels maintain their angiogenic potential**. **(A)** Representative images of the in situ hybridization performed with a DNA probe that stains human cellular nuclei (blue) in contrast with chicken nuclei (pink). Scale bar: 200 μm (left), 50 μm (right) **(B)** Quantification of the number human cells stained after ISH, after 4 days of implantation of prevascularized GGDVS-RGD spongy-like hydrogels after 14 days of preservation with HTS or α-MEM at 4 °C + 24h recovery at 37 °C in basal media. Data are presented as a violin plot illustrating the kernel density distribution frequency of the number of human cells and analyzed using one-way ANOVA with Tukey multiple comparison post-test (**p < 0.0021, and ***p < 0.0002). **(C)** Representative immunohistochemistry images of pre vascularized GGDVS-RGD spongy-like hydrogels after 4 days of implantation showing human CD31 positive cells (brown). Human CD31 expression pattern demonstrated the integration of the prevascular network in the CAM, before preservation (BP) and after preservation with HTS, as highlighted by black arrows Scale bar: 50 μm (left), and 20 μm (right). **(D)** Quantification of human CD31 stained area after immunohistochemistry, at 4 days of implantation of prevascularized GGDVS-RGD spongy-like hydrogels after 14 days of preservation with HTS or α-MEM at 4 °C + 24h recovery at 37 °C in basal media. Data are presented as a violin plot illustrating the kernel density distribution frequency of stained area and analyzed using one-way ANOVA with Tukey multiple comparison post-test (*p < 0.0332).

These findings suggest a beneficial role of prevascularization with SVF in construct integration and post-implantation vascularization, both mostly maintained when HTS was used as a preservation solution. The results of in situ hybridization revealed the persistence of human-origin cells from the SVF in the CAM tissue after 4 days of implantation (Fig. 6A). These cells were localized at the interface between CAM tissues and the implanted spongy-like hydrogels, albeit in a reduced number compared to the BP state (Fig. 6B). Additional analysis using immunohistochemistry for human CD31 revealed the existence of human-origin vessel-like structures at the interface between the CAM tissue and the implanted sponges (Fig. 6C). This was evident not only in the BP control group but also in the group preserved with HTS. While constructs preserved with α-MEM did retain some endothelial cells, the stained area corresponding to human CD31-positive cells was significantly less than the one found for constructs of both the BP and HTS groups (Fig. 6D). These cells were primarily confined within the implanted construct and were not located in the vicinity of vessel-like structures (Fig. 6C, Fig. S7A).

## 4 Discussion

Recent advancements in TE have led to the emergence of novel products aiming for market entry and application in patients as reviewed elsewhere [28,29]. Prevascularization is considered a critical feature in many of these strategies since it contributes to the in vivo survival and integration of TE products. Therefore, prevascularization must be a key concern of TE products preservation strategies. Existing limitations in preservation methods pose significant hurdles for the clinical implementation of these tissue engineering innovations, impacting their batch production, distribution, and adaptability to changing demand [7]. Preservation strategies for TE products, particularly for complex three-dimensional structures like those investigated here, are less explored compared to strategies targeting single cells or tissues/organs. To address this gap, short-term storage methods, such as hypothermia, have been explored to enhance the shelf-life of this products [[9], [10], [11]]. However, its impact on prevascular structures, a critical feature of many TE constructs, has not been studied. To the best of our knowledge, this is the first study specifically focusing on the mitigation of the effects of hypothermic preservation on prevascular structures. Considering the previously demonstrated potential of HTS for the preservation of certain TE product features such as cell survival and differentiation, the objective of this work was to assess its efficiency on the preservation of capillary-like structures within prevascularized tissue engineered constructs. To do this, a 3D prevascularized construct was generated by leveraging the angiogenic capabilities of SVF, and subsequently preserved at 4 °C, using either the preservation gold standard HTS or α-MEM as preservation media.

Angiogenic/vasculogenic signaling is pivotal for the prevascularization of engineered constructs. The presence of multiple cell types within the SVF allows for a dynamic regulation of angiogenic/vasculogenic-associated growth factor secretion [30]. The observed balance between ECM degradation, initially presenting a higher proteolytic activity by metalloproteinases (MMPs) and plasminogen activator (uPA) in earlier culture times, and the subsequent upregulation of factors like IL-8, MCP-1, thrombospondin-1 and VEGF associated with endothelial cell proliferation, migration, and organization over the course of culture, were crucial for the formation of a prevascular network [31]. The angiogenic stimuli provided by VEGF was further enhanced through the activation of downstream pathways associated with the αvβ3 integrin [32], including the transduction of angiogenic growth factors and tube formation [[33], [34], [35]]. Previous studies underscored the crucial significance of the interplay between SVF cells and RGD spongy-like hydrogels via the αvβ3 integrin, in orchestrating angiogenic events, especially by impacting the survival and migration of endothelial cells [6]. The observed interconnected microstructure of RGD spongy-like hydrogels alongside the inherent complexity of the SVF, and capacity to release diverse angiogenic growth factors, substantially contributed to the formation of an intricate and interconnected prevascular network, as evidenced by the expression pattern of CD31 after a span of 14 days.

Following the formation of a prevascular network, the engineered constructs were preserved under hypothermic temperatures for 14 days using either HTS or α-MEM as preservation solutions. At a temperature of 4 °C, cultured cells tend to adopt a more spherical shape as their internal structures undergo sequential disassembly, starting with microtubules and followed by microfilaments [37]. After exposure to 4 °C and re-incubation at 37 °C, cells typically regain their normal shape if any occurred damage is mild enough [36]. After the recovery period, cells preserved with HTS maintained their spindle-shaped morphology, indicating preserved structural integrity, whereas cells preserved with α-MEM exhibited a more rounded and fragmented morphology, suggesting inability of α-MEM to avoid disassembly of cytoskeleton and cellular damage, possibly leading to reduced viability. In fact, these morphological changes correlate with a noticeable decrease in DNA content observed during preservation with α-MEM. Interestingly, despite these observed differences in morphology and DNA content, there were no significant variations in the proportion of viable cells between the two preservation conditions compared to the baseline.

While it is widely acknowledged that in hypothermic preservation, a considerable amount of cell death occurs after rewarming, the exact underlying mechanism remains unclear. Hypothermia-induced injury can trigger both pro-survival and pro-apoptotic signals in cells [37,38]. Successful removal of damaged organelles, along with subsequent repair and adaptation, promotes cell survival. However, if the cell fails to restore homeostasis, they may undergo delayed apoptosis. During hypothermia, the increase in cytosolic Ca2^+^ levels induce stress in the endoplasmic reticulum and mitochondria, that is in turn amplified by the production of reactive oxygen species (ROS) [39]. The mitochondria are particularly vulnerable to this type of damage causing inner membrane permeabilization that culminates in mitochondrial depolarization, uncoupling of oxidative phosphorylation, and mitochondrial swelling [40]. When damage affects only a few mitochondria, it stimulates autophagy, leading to the lysosomal degradation of the affected mitochondria and removal of the signals that triggered autophagy [41,42]. However, when the level of stress becomes overwhelming, extensive autophagy can lead to cell death, possibly to maintain a significant level of ATP, which may be utilized for energy-consuming apoptotic processes [41]. Hence, apoptosis and autophagy can co-occur, as evidenced by their activation in constructs preserved with α-MEM. The simultaneous activation of both mechanisms indicates more significant cell damage. The use of nonspecific preservation solutions like α-MEM proves inadequate in counteracting the detrimental effects of hypothermia, especially concerning ROS scavengers and pH buffers. Unlike α-MEM, HTS possesses key attributes such as the ability to maintain ionic and osmotic balance, inhibit acidosis, prevent cell swelling, and notably, the presence of Trolox, a potent ROS scavenger [43,44]. Through the scavenging action of Trolox, the availability of ROS to inflict damage on organelles, particularly the mitochondria, is diminished. In this way, HTS manages to prevent the activation of various cellular stress pathways connected to oxidative stress, which in turn can lead to cell death. This is especially crucial for endothelial and immune cells, which are particularly sensitive to oxidative stress exposure. The reduction in DNA content and the decrease in the number of vessels, nodes, junctions, and meshes can be linked to the demise of these cells, considering their acknowledged susceptibility to ROS [[24], [25], [26], [27]]. Moreover, the observed reduction in the secretion of inflammatory and angiogenic factors, notably more pronounced in the α-MEM preserved condition, strengthens the idea of hypothermia deleterious effect on incorporated cells in the absence of proper counteracting solutions. It is possible that hASCs with greater resilience to hypothermic exposure are selectively favored. The observed increase in FGF-b levels, especially in the α-MEM condition, suggests the potential reduction of SVF heterogeneity and the preferential selection of more stress-resistant hASCs. Additionally, considering hASCs are known to produce higher quantities of this cytokine compared to whole SVF [45], it is plausible that hASCs benefit from its significant role in the self-renewal process, including cell proliferation and differentiation [46].

This hypothesis was supported after the implantation of the constructs within a CAM model. The existence of a more diverse cell population, characterized by a robust 3D prevascular network both BP and after preservation with HTS, facilitated the infiltration of host cells into the implanted construct, without an exacerbated inflammatory response. The presence of a preexisting 3D prevascular networks before implantation is believed to accelerate the in vivo vascularization process of graft [47]. The demonstration of implanted human cells, particularly CD31-positive cells, integrating into freshly formed blood vessels at the interface between the CAM and the implanted construct BP and preserved with HTS, strongly supports this hypothesis. Conversely, in the case of preservation with α-MEM, while the presence of CD31-positive cells at the implantation site is evident, they are primarily confined within the implanted construct and couldn't be found in the vicinity of vessel-like structures. Results suggest that the deleterious effects of hypothermia on SVF constructs, left unchecked by α-MEM and leading to the disruption of the prevascular network, resulted in reduced ingrowth of host cells after implantation and, therefore, in decreased construct integration when compared with fresh SVF constructs or SVF constructs preserved with HTS. Nevertheless, when compared with the acellular construct, SVF constructs preserved with α-MEM had better integration, suggesting that even in sub-optimal conditions, implanted SVF can have a positive effect on construct integration [[48], [49], [50]].

While encouraging, it's important not to regard the presented strategy as a panacea for every other prevascularized TE product. The preservation of such products requires a customized approach, recognizing their diverse nature, namely in terms of engineered tissue, volume, etc., rather than relying on a universal strategy. In fact, customization will require in-depth analysis of the effects of the specific hypothermic protocol in each of the cell components of the construct. The lack of such an analysis can be regarded as a limitation of the present work. In future studies, more comprehensive cellular and molecular characterization methodologies, such as, for e.g., single cell RANseq, will be used to obtain specific insights into the cells more affected during hypothermic preservation. This is important when utilizing a mixture of cells such as SVF and will be even more critical when vasculogenic cells are mixed with other tissue-specific cells. Furthermore, the transition to the in vivo setting will introduce additional variables and complexities that may complicate implementation. The utilization of a CAM model in our study, while insightful, requires careful consideration when extrapolating these findings directly to human clinical applications. This is inherently an early screening model, undoubtedly superior to in vitro models but still with significant limitations. To enhance the translational relevance of the observed outcomes in the context of human TE, future investigations employing more clinically relevant models are imperative.

## 5 Conclusions

This study demonstrated that hypothermic preservation using a specialized solution such as HTS maintained cell viability and preserved the established prevascular network in an engineered construct, known to be crucial for in vivo performance after implantation. The action of HTS was essential in preserving the positive effect of SVF on construct integration and vascularization in a CAM model, including the incorporation of implanted human cells into newly formed blood vessels. Overall, this research highlights, on the one hand, the huge potential of SVF to improve in vivo integration and vascularization of engineered constructs and, on the other, the potential of a tailored preservation solution like HTS to effectively preserve the potential of SVF, mitigating hypothermia-induced cell damage and offering a valid solution for TE products transportation and storage.

## Ethics approval and consent to participate

All authors declare that this manuscript is original, has not been published before and is not currently being considered for publication elsewhere. The manuscript has been read and approved by all named authors and all agree with its submission to the journal Bioactive Materials.

## CRediT authorship contribution statement

**Sara Freitas-Ribeiro:** Writing – review & editing, Writing – original draft, Methodology, Investigation, Formal analysis, Data curation, Conceptualization. **Helena Moreira:** Resources. **Lucília P. da Silva:** Writing – review & editing, Resources, Investigation, Formal analysis. **Jennifer Noro:** Investigation, Formal analysis. **Belém Sampaio-Marques:** Writing – review & editing, Methodology, Investigation, Formal analysis. **Paula Ludovico:** Writing – review & editing, Resources. **Mariana Jarnalo:** Resources. **Ricardo Horta:** Resources. **Alexandra P. Marques:** Writing – review & editing, Validation, Data curation. **Rui L. Reis:** Writing – review & editing, Validation, Supervision, Funding acquisition. **Rogério P. Pirraco:** Writing – review & editing, Writing – original draft, Validation, Supervision, Methodology, Funding acquisition, Data curation, Conceptualization.

## Declaration of competing interest

None.
