# Supplementary material for: Prevascularized spongy-like hydrogels maintain their angiogenic potential after prolonged hypothermic storage
Source: Bioact Mater. 2024 Mar 28;37:253–68. doi: 10.1016/j.bioactmat.2024.02.035 (PMC10997873; doi:10.1016/j.bioactmat.2024.02.035)
Supplement: Multimedia component 1 [file mmc1.docx]

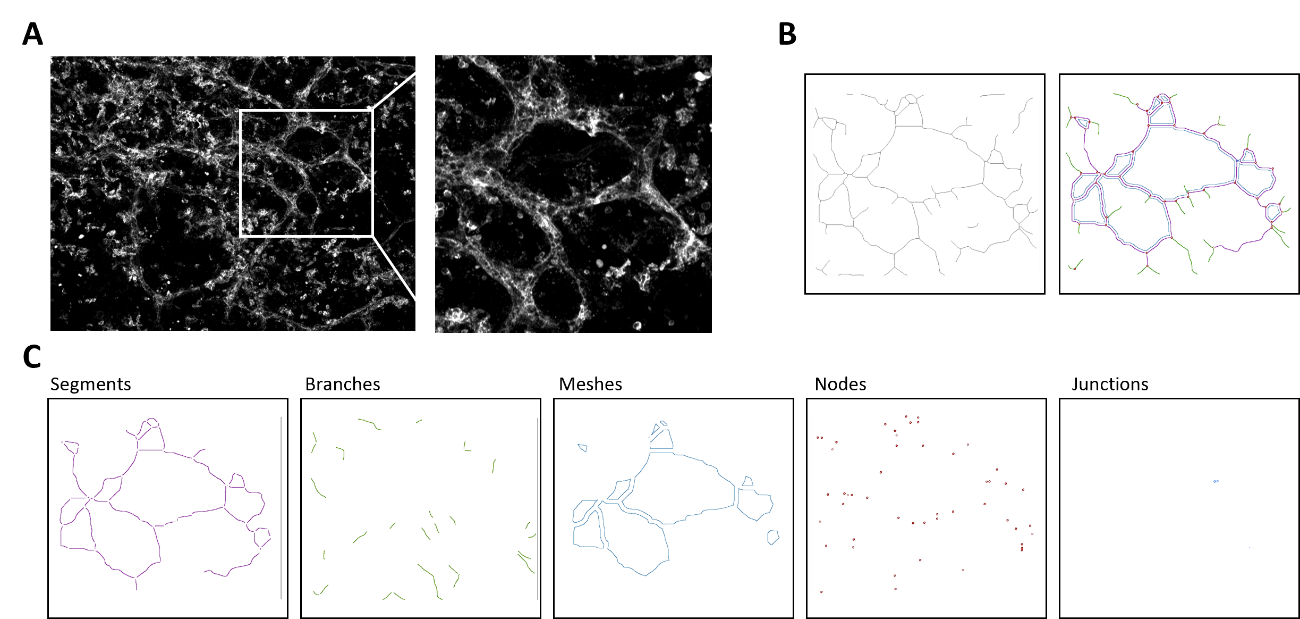


**Fig. S1. Schematic representation of the measurements obtained from the Angiogenesis Analyzer in ImageJ.** **A)** Initial image used for the measurements. **B)** Skeleton of the image. **C)** Detection of segments (purple) – lines connected by two nodes, branches (green) – lines connected by a node, meshes (ligh blue) – closed areas surrounded by segments, nodes (red) – minimum structure allowing a bifurcation, and junctions (dark blue) – four of more connected segments.


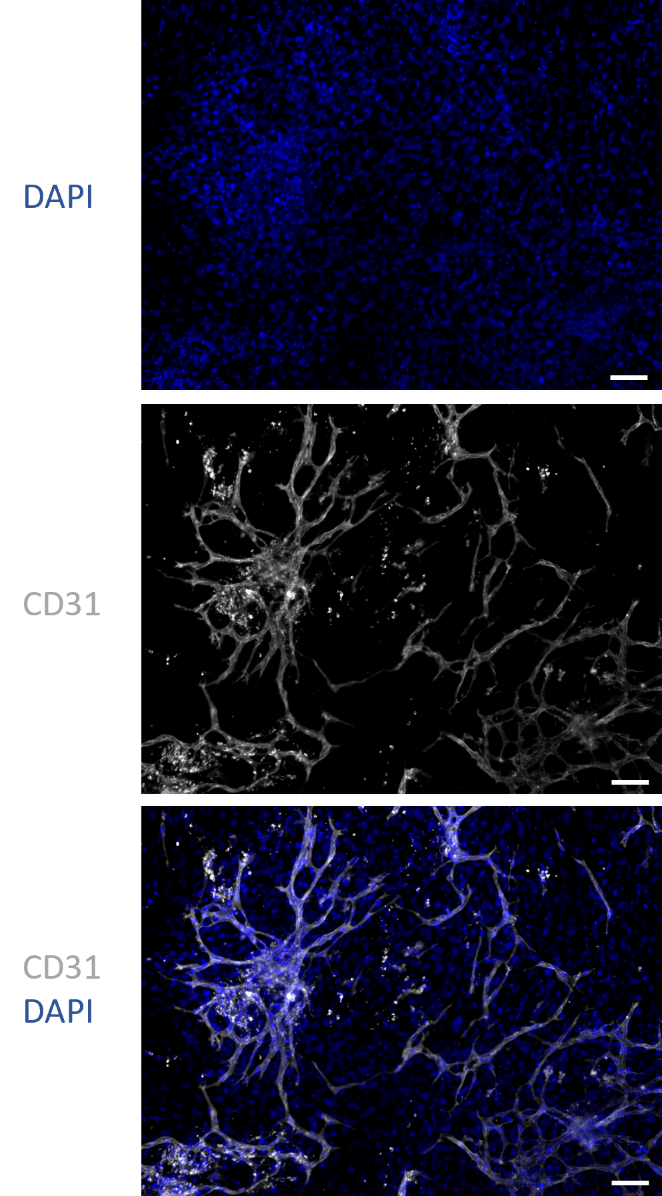


**Fig. S2.** Representative immunocytochemistry images of the organization of SVF-derived CD31 (white) expressing cells in 2D conditions on standard TCPS. Scale bar: 100 µm.


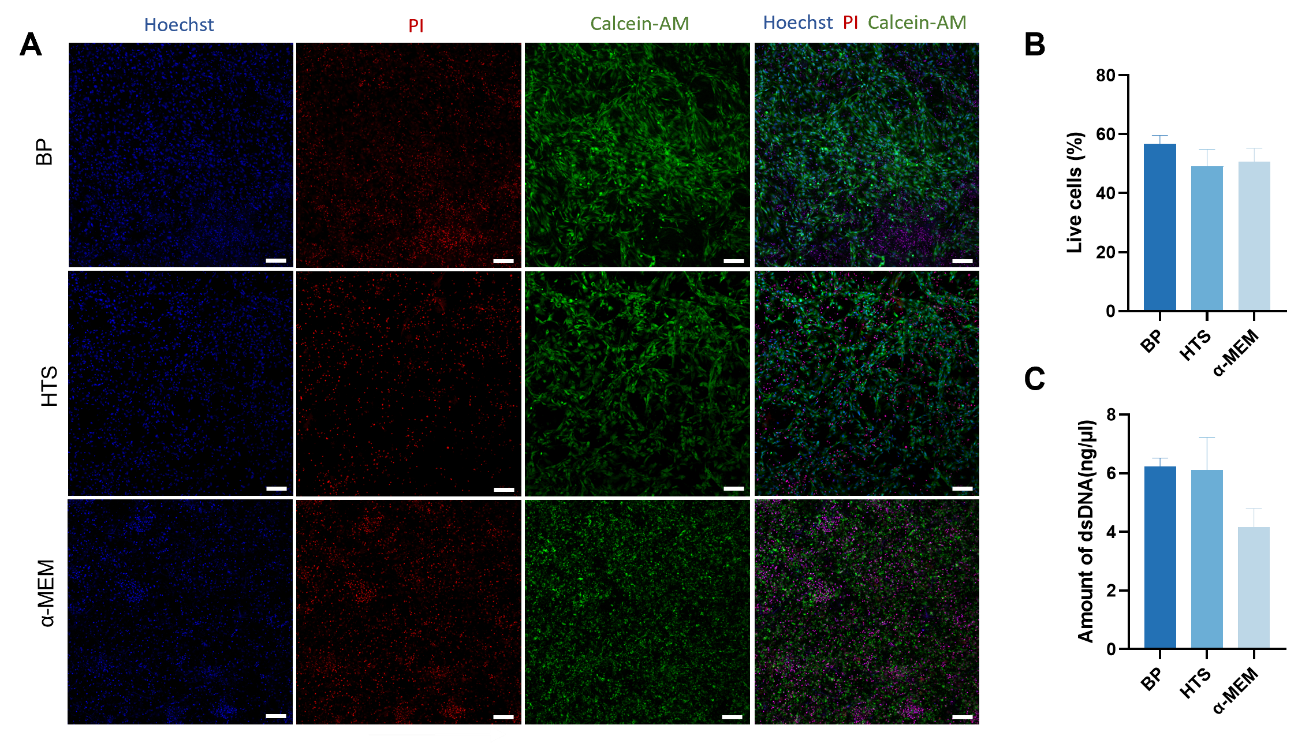


**Fig. S3. Preserved prevascularized spongy-like hydrogels viability. (A)** Representative images of viability staining, **(B)** Quantification of % live cells and **(C)** Amount dsDNA of pre vascularized GGDVS-RGD spongy-like hydrogels before preservation (BP) and after 14 of preservation with HTS and α-MEM at 4ºC + 24h recovery at 37ºC in basal media. Viable cells were stained with Caelcein-AM (green) and dead cells stained with PI (red). Cell nuclei were counterstained with hoechst (blue).


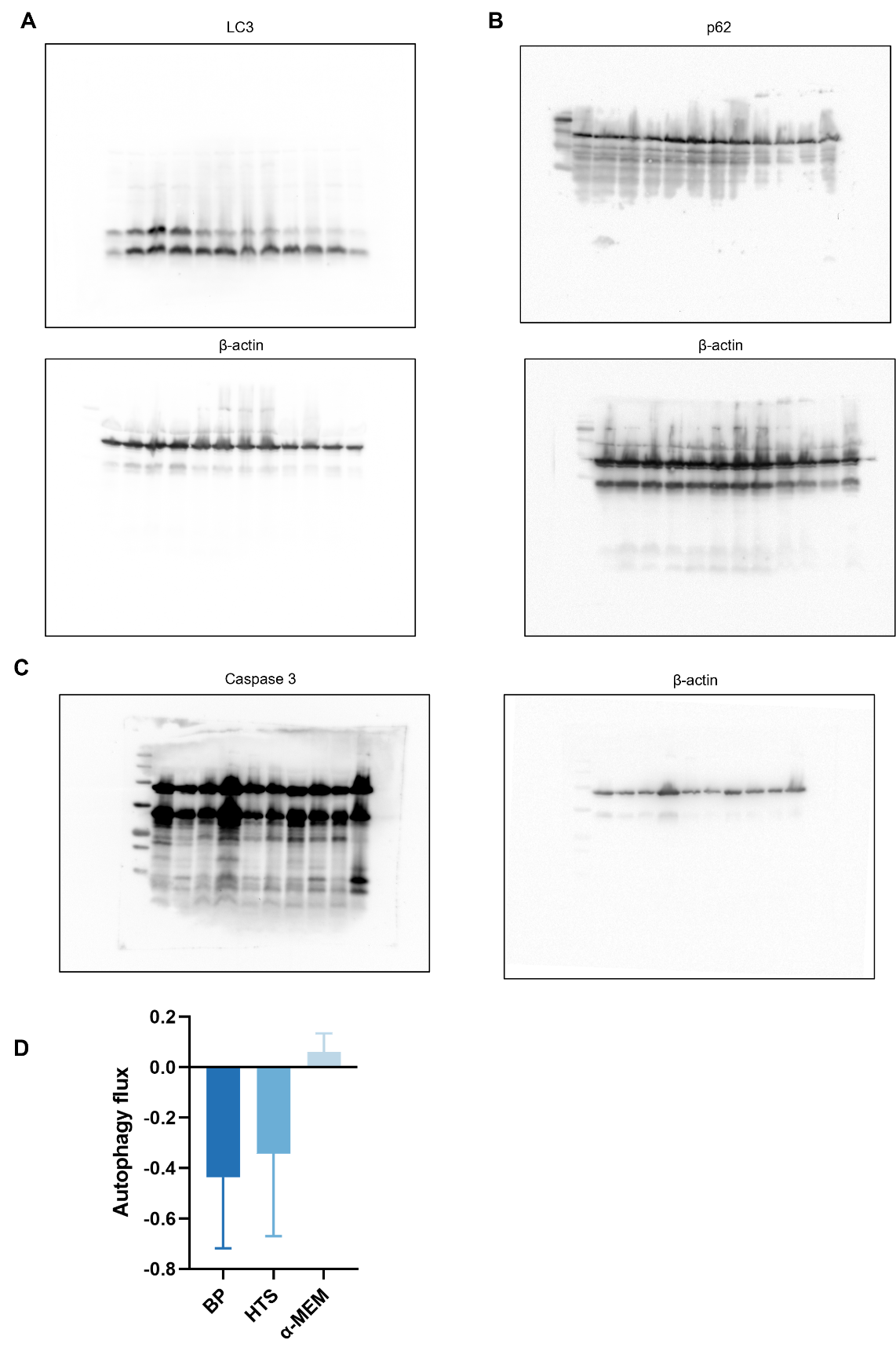
**Fig. S4.** Original western blot membranes of **A)** LC3 I and II and respective β-actin, **B)** p62 and respective β-actin and **C)** Caspase 3 and respective β-actin, **D)** Autophagy flux quantification. Autophagy flux was assessed by comparing relative LC3-II levels under autophagy induction with metformin against basal conditions for each preservation condition or before preservation. A positive flux denotes autophagy induction, while a negative flux indicates a blockage in the process.


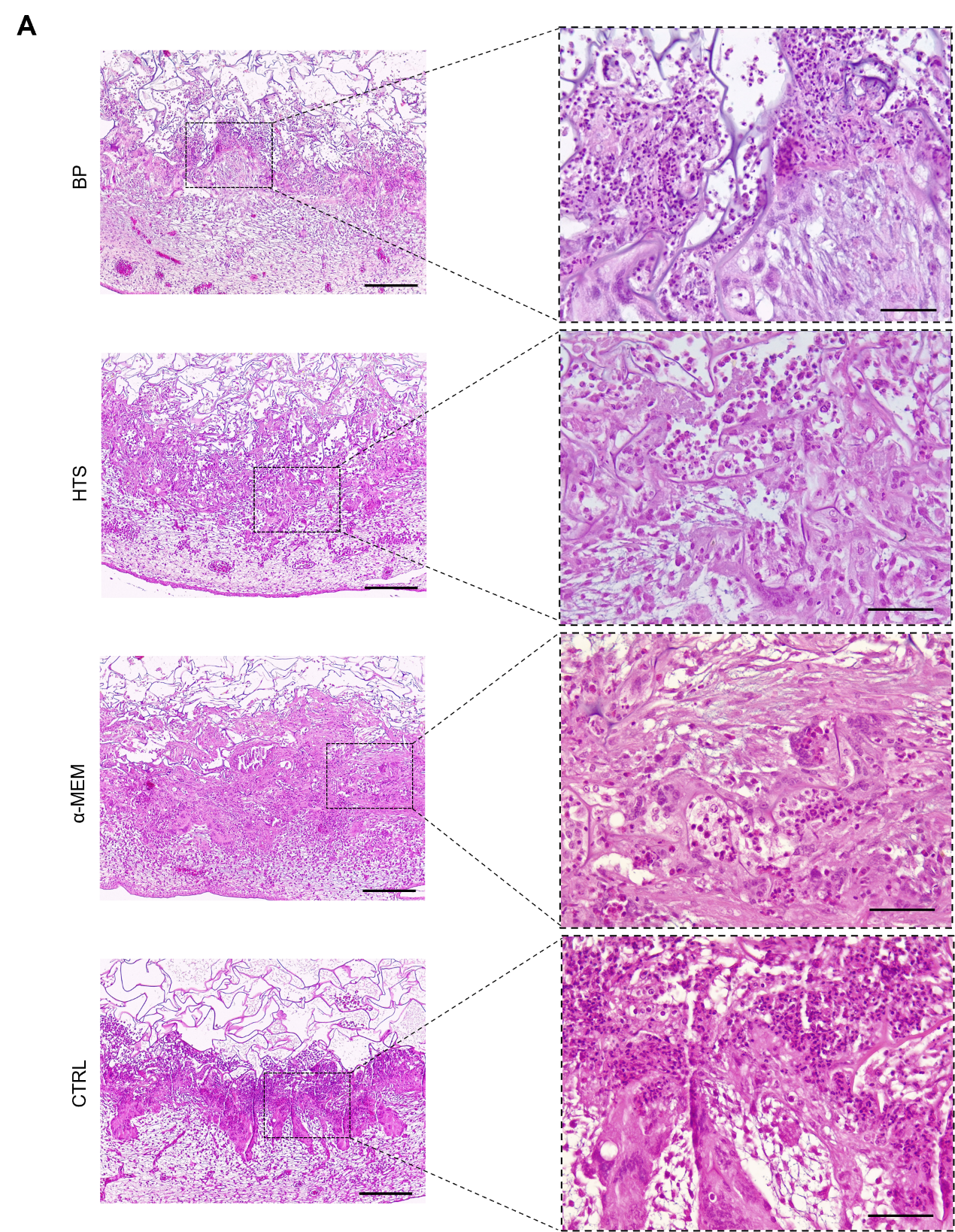
**Fig. S5. A)** Representative micrographs of hematoxylin and eosin staining in pre vascularized GGDVS-RGD spongy-like hydrogels before preservation (BP) and after 14 days of preservation with HTS and α-MEM at 4ºC + 24h recovery at 37ºC in basal media. Scale bar: 200 µm (left), 50 µm (right)


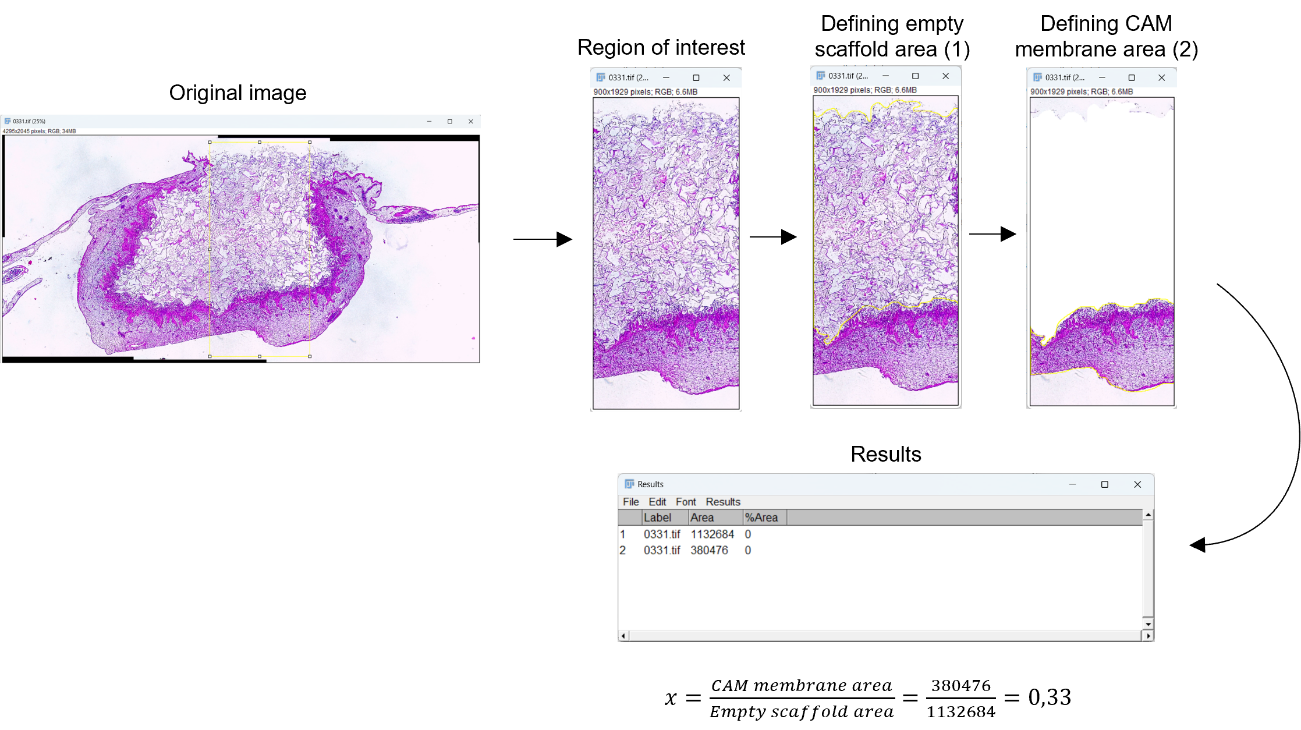
**Fig. S6. A)** Schematic representation of the measurements obtained from representative micrographs of hematoxylin and eosin staining in ImageJ. First, a region of interest, with a width of 900 pixels and a height determined by the construct, was defined for each image. After defining the area of non-integrated “empty” scaffold in the image, the corresponding region was measured. The same procedure was performed for the CAM membrane area. Finally, the membrane-scaffold ratio was obtained using the provided formula.


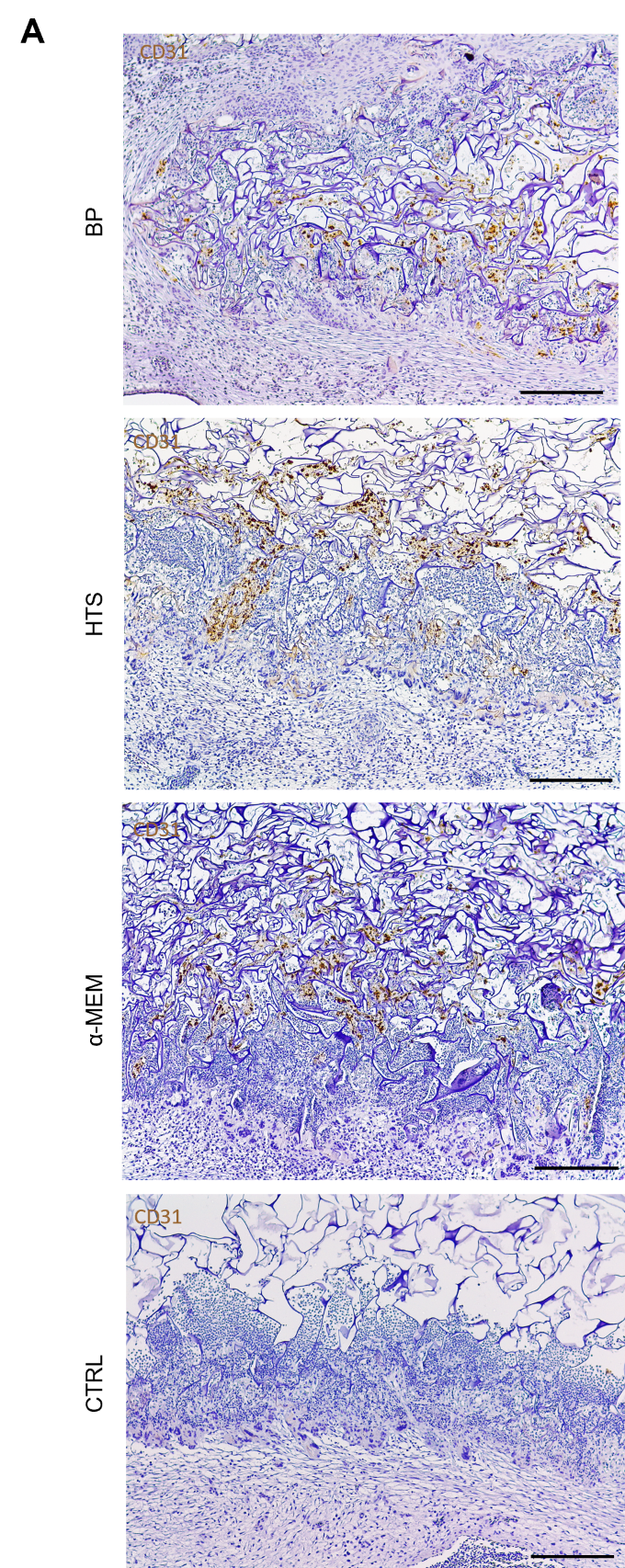
**Fig. S7. A)** Representative immunohistochemistry images of pre vascularized GGDVS-RGD spongy-like hydrogels after 4 days of implantation showing human CD31 positive cells (brown). Scale bar: 200 µm.
